# Short-Term Outcomes and Clinical Efficacy of Stereotactic Body Radiation Therapy (SBRT) for Oligometastases of Prostate Cancer in China

**DOI:** 10.3389/fonc.2022.879310

**Published:** 2022-04-28

**Authors:** Chenyang Xu, Xianzhi Zhao, Xiaoping Ju, Yuxin Shen, Min Qu, Yusheng Ye, Xiaoyan Wang, Chunshan Yu, Xu Gao, Huojun Zhang

**Affiliations:** ^1^ Department of Urology, Huashan Hospital, Fudan University, Shanghai, China; ^2^ Department of Radiation Oncology, Shanghai Changhai Hospital, The Navy Medical University, Shanghai, China; ^3^ Department of Urology, Shanghai Changhai Hospital, The Navy Medical University, Shanghai, China

**Keywords:** oligometastases, stereotactic body radiotherapy (SBRT), metastatic castration-resistant prostate cancer (mCRPC), metastatic hormone-sensitive prostate cancer (mHSPC), efficacy

## Abstract

**Objective:**

To assess the efficacy and safety of stereotactic body radiation therapy (SBRT) in managing oligometastases of prostate cancer. Moreover, it is the largest-to-date study in China to report the safety and efficacy of SBRT by CyberKnife for oligometastases of prostate cancer.

**Methods:**

In this retrospective study, 75 patients with 108 oligometastases were treated by SBRT from May 2012 to February 2021. Among these patients, 43 patients were treated with the intention to control all known metastatic lesions and 32 were treated for palliative care. Patients received regular follow-up evaluations every 3 months. Efficacy was assessed based on local control (LC) rates, biochemical progression-free survival (bPFS), progression-free survival (PFS), and overall survival (OS). Safety was assessed based on clinical adverse events.

**Results:**

Median follow-up time was 23.2 months (1.2-106.9 months). The complete response (CR), partial response (PR), stable disease (SD), and progressive disease (PD) rates were 63.0%, 10.2%, 21.3% and 5.6%, respectively. The 6-month, 1-, and 2-year LC rates were 100%, 97.5%, and 96.0% respectively while the 6-month, 1-, and 2-year bPFS rates were 74.6%, 53.3%, and 47.9%, respectively. Additionally, 6-month, 1-, and 2-year PFS rates were 77.5%, 50.8%, and 47.2%, respectively. The 6-month, 1-, and 2-year OS rates were 97.0%, 88.8%, and 87.0%, respectively. For the 15 metastatic castration-resistant prostate cancer (mCRPC) patients with 23 lesions, the 2-year LC rates were 93.8%, while for 60 metastatic hormone-sensitive prostate cancer (mHSPC) patients with 85 lesions, the 2-year LC rates were 96.7%. No predictors of LC were found after univariate analysis. In those not on androgen deprivation therapy (ADT; n = 27), the 2-year freedom from ADT was 44.0%. All of the 24 patients with oligmetastase-induced complications experienced varying degrees of alleviation after SBRT. The treatment was well tolerated. No grade 3 or higher toxicity was observed.

**Conclusion:**

SBRT is a safe and effective treatment modality in the management of oligometastases of mHSPC and mCRPC with high LC rates and acceptable toxicity. SBRT could provide a treatment choice for mCRPC, as well as an alternative to delay the start of ADT for mHSPC.

## Introduction

Prostate cancer (PCa) is one of the most common genitourinary malignancies worldwide. The incidence and mortality of PCa has been increasing in China in the past decades. Metastatic PCa occurred in one-third of the patients after primary treatment (1). Systemic treatment for metastatic PCa was necessary, especially in patients with intermediate and high-risk of progression. Management options included androgen deprivation therapy (ADT), abiraterone, chemotherapy, immunotherapy, etc. (2, 3). Metastases-directed treatment included salvage surgery, external-beam radiotherapy, and brachytherapy, which may facilitate local control of metastatic lesions, relieve symptoms, and delay systemic treatment (4). However, the results have not been satisfactory including failure of tumor control, adverse reactions, and castration resistance. Therefore, exploration of more effective treatment to prolong tumor control and minimize toxicity is a much discussed topic.

Oligometastatic PCa is commonly proposed as an interim state between localized PCa and widely-spread PCa. In recent years, stereotactic body radiation therapy (SBRT) has emerged as one of the promising metastases-directed treatment options for oligometastatic malignancies. SBRT can be performed with a conventional linear accelerator or CyberKnife. Compared with conventional linear accelerator, CyberKnife has a real-time tracking system that can correct the beam angle by identifying the patient’s breathing patterns, which is a huge innovation (5).

Several randomized phase 1/2 trials suggested the safety and potential benefits of SBRT for oligometastatic PCa. Compared with active surveillance, SBRT could prolong ADT-free survival (21 months vs 13 months). Quality of life (QoL) was similar between the two groups and no grade 2-5 toxicity was reported in a median follow-up time of 3 years (6). One recent phase 2 trial compared progression at 6 months between SBRT and observation in 54 metastatic PCa patients after randomization in a 2:1 ratio. Progression (defined as prostate-specific antigen level increase, progression detected by conventional imaging, symptomatic progression, ADT initiation for any reason, or death) rate at 6 months were 19% vs 61% (P=0.005) between the two groups. These studies provided preliminary evidence for application of SBRT in metastatic PCa (7). However, limitations of these studies include relatively small sample size and lack of long-term follow-up results.

Thus, the role of SBRT as a metastases-directed therapy for oligometastases remained to be explored. The aim of this real-world analysis was to assess the efficacy and safety of SBRT by CyberKnife for oligometastatic PCa.

## Material and Methods

### Participants

We reviewed all the oligometastatic PCa patients treated with SBRT at any line at First Affiliated Hospital of Navy Medical University. All these patients were examined by an oncologist to confirm the diagnosis of metastatic PCa before the treatment. Patients with oligmetastases (no more than 5) diagnosed by imaging examinations (e.g. MR, Bone scan, FDG PET/CT, PSMA PET/CT, or PSMA PET/MR), a Karnofsky performance score no less than 70, a life expectancy of over 3 months were included in the study. Patients who declined SBRT or were unsuitable for SBRT due to comorbidities were excluded. Patients were also excluded if the metastatic lesion had been previously treated by radiotherapy. Informed consents were obtained from all patients prior to the enrollment and the study was conducted according to the Declaration of Helsinki. The study protocol was reviewed and approved by the Medical Ethics Committee of First Affiliated Hospital of Navy Medical University. In total, 75 patients with oligometastases of PCa (total 108 lesions) between May 2012 and February 2021 constituted the dataset.

### Treatments

Of the 75 patients, 43 were treated with the intent to control all known metastatic lesions, and 32 underwent SBRT for palliation of oligometastases. SBRT was delivered by CyberKnife (Accuray Corporation, Sunnyvale, CA, USA). Patients were immobilized in supine position with arms by their sides using thermoplastic body mask. Enhanced computed tomography (CT) scan was performed with a slice thickness of 1.5** mm**, with the scan range of at least 10** cm** below and above the tumor. The gross tumor volume (GTV) was defined as a radiographically lesion in the oligometastases. According to the metastases motion, planning target volume (PTV) was delineated with a 2-6** mm** margin expansion in lateral direction and in anteroposterior direction respectively, a 2-8** mm** margin expansion incephalo-caudal direction from GTV. For 68 patients, X-sight spine tracking was used, while 4 patients with synchrony respiratory motion tracking and 3 patients with 6D-skull tracking. X-sight spine tracking was employed for SBRT in 68 patients with 101 lesions, while synchrony respiratory motion tracking was performed in 4 patients with 4 lesions and 6D-skull tracking in 3 patients with 3 lesions. The dose-volume constraints for organs at risk were referred to the American Association of Physicists in Medicine guidelines in TG-101 (8).

### Outcome Measurements and Follow-Up

The primary outcome of efficacy was local control (LC) rate. Secondary outcomes include biochemical progression-free survival (bPFS), progression-free survival (PFS), overall survival (OS), and adverse events (AE). Serum prostate-specific antigen (PSA) and/or testosterone levels of the patients were checked every month. Biochemical failure was defined as (1) in the case of the initial decline from baseline after SBRT, the first PSA increase that was 25% and 2 ng/ml above the nadir, or an increase that was 25% and greater than the pre-treatment PSA value, as confirmed by a second value 3 or more weeks later; or (2) in the case of no initial decline from baseline, a PSA increase that was 25% and 2 ng/ml greater than baseline after 3 months if baseline PSA was 2 ng/ml, or PSA increase that was 25% after 3 months if baseline PSA was <2 ng/ml (9).

Contrast-enhanced CT scans, SPECT, 68-Ga Prostate-specific membrane antigen (PSMA) PET/CT scans, or contrast-enhanced MRI was performed every 3 months after radiotherapy to monitor recurrence or progression. Adverse events, amelioration of symptoms and sequential treatment were recorded. Acute and late toxicity were scored according to the Common Terminology Criteria for Adverse Events (CTCAE) version 6.0. LC was defined as complete response (CR), partial response (PR), and stable disease (SD). Tumor response was determined using the Response Evaluation Criteria in Solid Tumors (RECIST), version 1.1 (10). OS was defined as the time from the start of SBRT to the death of any cause or the last follow-up. PFS was defined as the time from the start of SBRT to the confirmation of disease progressions at any site or death by any cause.

### Statistical Analysis

The curves of LC, bPFS, PFS, and OS were calculated by the Kaplan-Meier method. Potential factors associated with LC rate were identified with univariate log-rank comparisons. Statistical analyses were performed using SPSS 18.0 (IBM Corporation, Armonk, NY, USA). Two-sided P values **<**0.05 were considered statistically significant.

## Results

### Patient Characteristics

Basic characteristics of the 75 patients were analyzed. The median age of study cohort was 68 years, ranging from 51 to 88 years. The median PSA at PCa diagnosis and before SBRT for oligometastases was 44.7 ng/m and 4.5 ng/ml, respectively. Nearly two-thirds of the patients (49/75) had primary tumor with Gleason score 8 or higher. Among the 108 metastases, 12.0% (13/108) were lymph node metastases (N1 or M1a), 82.4% (89/108) were bone metastases (M1b), and 5.6% (6/108) had visceral metastases (M1c). Twenty-three patients (30.7%) had more than one metastatic lesion. According to the CHAARTED criteria (11), 8.0% (6/75) of the patients had high metastatic burden. Median time duration between PCa diagnosis and oligometastases diagnosis was 30.1 months (range 11.6-45.8 months). While median time duration between oligometastases diagnosis and SBRT was 1.4 months (range 0.5-5.5 months). Detailed information of patient characteristics was is in **Table 1**. The treatment parameters are presented in **Table 2**.

**Table 1:** Patient characteristics.

Characteristics	All patients (N=75)
Age at SBRT-year	68 (range 51-88)
Karnofsky performance score ≥70	75 (100%)
PSA at primary diagnosis-ng/ml	44.7(3.4-999.9)
PSA before SBRT-ng/ml	4.5 (0.001-999.9)
Gleason Score	
6	1 (1.3%)
7	20 (26.7%)
8	16 (21.3%)
9	28 (37.3%)
10	5 (6.7%)
N/O	5 (6.7%)
No. of metastatic lesions at SBRT	
1	52 (69.3%)
2	16 (21.3%)
3	5 (6.7%)
4	1 (1.3%)
5	1 (1.3%)
Number of metastases	
Lymph node metastases	
Regional lymph nodes (N1)	6 (5.6%)
Non-regional lymph nodes (M1a)	7 (6.5%)
Bone metastases (M1b)	
Axial	60 (55.6%)
Appendicular	29 (26.9%)
Visceral metastases (M1c)	
Lung metastases	3 (2.8%)
Brain metastases	1 (0.9%)
Adrenal gland metastases	2 (1.9%)
Castration-sensitivity before SBRT	
mCRPC	15 (20%)
mHSPC with ADT	33 (44%)
mHSPC without ADT	27 (36%)
Diagnosis time of oligometastases	
Primary oligometastatic prostate cancer	29 (38.7%)
Relapse after radical surgery	35 (46.7%)
Relapse after radical RT	11 (14.7%)
Imaging modality at recurrence	
F-18 FDG PET/CT	21 (28.0%)
Ga-68 PSMA-PET/CT	23 (30.7%)
Ga-68 PSMA-PET/MR	11 (14.7%)
SPECT	11 (14.7%)
MR	9 (12.0%)
Time from primary diagnosis to metastases-month	12.8 (0.0-122.1)
Time from metastases to SBRT-month	1.6 (0.0-58.8)

SBRT, Stereotactic body radiation therapy; PSA, Prostate specific antigen; N/O, Not obtained; mCRPC, Metastatic castration-resistant prostate cancer; mHSPC, Metastatic hormone-sensitive prostate cancer; ADT, Androgen deprivation therapy; RT, radiation therapy; SPECT, Single-Photon Emission Computed Tomography.

**Table 2:** Treatment parameters for SBRT.

Parameters	Median (Range)
GTV (ml)	7.8 (0.5-83.3)
Total prescribed dose (Gy)	33.6 (16 - 45)
Number of fractions	5 (4 - 8)
Dose per fraction (Gy)	6.5 (4 - 8)
BED_1.5_ (Gy)	170.9 (58.7-270.0)
Maximum dose (Gy)	46.3 (23.3-69.7)
Prescription isodose line (%)	72 (61 – 89)

GTV, Gross tumor volume; BED_1.5_, Biologic equivalent dose (α/β=1.5Gy).

### Efficacy Outcomes

**Figure 1:**
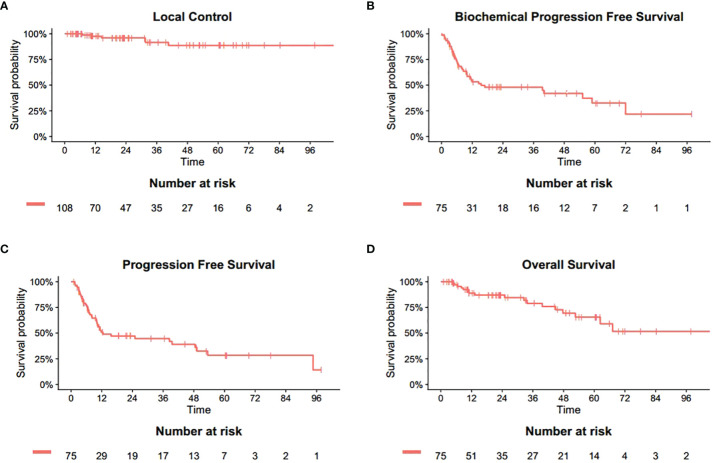
Actuarial survival analysis of patients. **(A)** Overall local control. **(B)** Overall biochemical progression free survival. **(C)** Overall progression free survival. **(D)** Overall survival.

The median follow-up duration after SBRT was 23.2 months (range 1.2-106.9 months). The 6-month, 1-, 2-year LC rates were 100%, 97.5%, 96.0%, respectively (**Figure 1A**). Based on the RECIST criteria, the CR, PR, and SD rates were 63.0%, 10.2%, and 21.3% respectively, while six (5.6%) lesions of five patients had disease progression (PD) among the 108 metastatic lesions after SBRT. Detailed information is shown in **Table 3**. For detailed information of local progressive disease, see **Table S1**. For the 15 metastatic castration-resistant prostate cancer (mCRPC) patients with 23 lesions, the 2-year LC rate was 93.8% while for 60 metastatic hormone-sensitive prostate cancer (mHSPC) patients with 85 lesions, the 2-year LC rate was 96.7%. In the univariate analysis, mCRPC patients after SBRT had a similar LC rate with those mHSPC patients (P=0.898). Similarly, no other predictors was associated with LC after univariate analysis (**Table 4**). In those not on ADT (n= 27), the 2-year freedom from ADT was 44.0%. Among the patients who had olgmetastases-induced symptoms prior to the treatment (including 21 with corresponding pain and 3 with physical weakness), all of them (100.0%) had varying degrees of alleviations of symptoms after SBRT.

**Table 3:** Local control following SBRT.

Metastatic sites	CR	PR	SD	PD
Lymph node (N=13)	10	–	3	–
Pelvic (N=6)	3	–	3	–
Extrapelvic (N=7)	7	–	–	–
Bone (N=89)	55	9	19	6
Axial (N=60)	36	6	13	5
Appendicular (N=29)	19	3	6	1
Visceral (N=6)	3	2	1	–
Lung (N=3)	2	1	–	–
Adrenal gland (N=2)	1	1	–	–
Brain (N=1)	–	–	1	–

CR, complete response; PR, Partial response; SD, Stable disease; PD, Progressive disease.

For detailed information of progressive disease, see **Table S1**.

**Table 4:** Univariate analysis for LC rate.

	1-year LC rate (%)	2-year LC rate(%)	P Value
Castration-sensitivity			
mHSPC	98.4	96.7	0.898
mCRPC	93.8	93.8	
BED_1.5_ (Gy)			
<180	96.5	94.4	0.104
≥180	100	100	
GTV(ml)			
<20	100	100	0.053
≥20	88.4	81.1	
Systemic therapy after SBRT			
Yes	97.0	95.3	0.300
No	100	100	

LC, local control; mCRPC, Metastatic castration-resistant prostate cancer; mHSPC, Metastatic hormone-sensitive prostate cancer; BED_1.5_, Biologic equivalent dose (α/β=1.5Gy); GTV, Gross tumor volume; SBRT, Stereotactic body radiation therapy.

The 6-, 12- and 24-month bPFS was 74.6%, 53.3% and 47.9%, respectively (**Figure 1B**). The results of PFS were similar. The 6-, 12- and 24-month PFS was 77.5%, 50.8% and 47.2%, respectively (**Figure 1C**). Median time to distant progression was slightly longer than biochemical failure (25.1 month vs 24.9 month). Meanwhile, a total of 48 patients experienced distant progression. Most newly discovered metastases involved single organ such as bone, lymph node, and lung, which were treated with hormone therapy, chemotherapy, radiation therapy, or combination therapy.

At the last follow-up, 18 patients (24.0%) died while 57 were alive. One patient died of pneumonia and renal failure, respectively, whereas 16 patients died of distant metastasis. Hence, local failure and radiation-induced toxicity did not contribute to the deaths. The 6-month, 1-, 2-year OS was 97.0%, 88.8%, 87.0%, respectively (**Figure 1D**).

### Toxicity

SBRT was well-tolerated in PCa patients with oligometastases. No Grade 3 or higher adverse events were reported. Early toxicities after treatment included fatigue, nausea, decreased appetite, and leucopenia. Late complications included localized fibrosis, urinary frequency, etc. No fracture was observed during follow-up. All the early adverse effects were temporary, and cured by symptomatic medication.

## Discussion

This study evaluated the efficacy and safety of SBRT in oligometastatic PCa with lesions up to five. In a median follow-up duration of nearly 2 years, SBRT provided survival benefits with high LC rates. No severe adverse events (grade 3 or more) were reported.

Efficacy of SBRT in oligometastatic PCa has been evaluated in several studies. One prospective, single institutional clinical trial recruited 199 patients with relapsing oligometastatic PCa (lesions up to five) following definitive local treatment for primary PCa. After SBRT (50 Gy in 10 fractions) to each visible lesion, the median treatment escalation-free survival was 27.1 months, with 51.7% of the patients requiring no treatment escalation 2 years following SBRT (12). Apart from prolonging treatment escalation-free survival, SBRT may improve quality of life (QoL) because of a delay of more toxic salvage therapies (13). In a prospective clinical trial, stereotactic ablative body radiotherapy (SABR) for oligometastatic PCa in 22 patients not on ADT, the 2-yr freedom from ADT was 48.0% (14). Similarly, 27 patients were not on ADT in our study, and the 2-year freedom from ADT was 44.0%. In a large international study cohort of 1033 patients with extracranial oligometastases, SBRT provided favorable long-term OS and wide-spread progression rates, especially in the PCa patients (132 cases). The 3-year OS rate was 87.9% in the patients with PCa oligometastases (15), while 2-year OS rates were 87.0% in our study.

Another study included 64 oligorecurrent or oligoprogressive PCa (lesions up to five) and the median follow-up was 15.2 months. Rates of LC at 6-, 12- and 18-months were 94%, 88% and 84%. In the study cohort, CRPC patients had worse PFS compared with HSPC patients (16). However, in our study, mCRPC patients after SBRT had a similar LC rate as those with mHSPC (P=0.898). The 6-month, 1-, and 2-year LC rates were 100%, 97.5%, and 96.0%, respectively, which was higher than in the study mentioned above. It could be possible that combination use of SBRT and novel anti-androgen agents (e.g. Arbiraterone) led to this result for mCRPC patients (17). Ongoing trials are focusing on the combination of SBRT and other treatments in the scenario of oligometastatic CRPC (e.g., NCT02816983, NCT03503344, NCT03449719). The result of our study would contribute to this particular scientific interest, especially in the Chinese population.

Different metastatic patterns might not influence efficacy of SBRT. Bone and lymph node metastases were the most common in PCa patients. A multi-institutional study reported clinical data of 74 PCa patients with bone-only oligometastases (lesions up to 5). The 2-year PCa-specific survival (PCSS) and PFS rates were 92.0% and 72.0%, with LC rate of 95.4% per lesion. Single oligometastases and PSA response were associated with better PCSS and PFS in the multivariable analysis (18). A phase 2 trial evaluated high-dose SBRT for patients with lymph node oligometastases (lesions up to 3), most of whom were PCa patients. The OS at 1, 2, and 3 years were 97.3%, 94.2%, 84%, and PFS at 1, 2, and 3 years were 67.4%, 49.6%, and 46.1%. SBRT was well-tolerated among these patients (19). Another prospective phase 2 trial reported 5-year OS, PFS, and bPFS to be 96.9%, 88.2%, and 91.4% in 44 patients with locally advanced, node-positive, and bone oligometastatic PCa, using extreme hypofractioned radiation therapy (20). The similar results were observed in our study.

Combining other treatment modalities might improve the efficacy of SBRT. For instance, concurrent sunitinib and SBRT significantly improve the overall survival of PCa oligometastases (HR = 0.25, p = 0.04) (21). Combining cytoreductive prostatectomy and SBRT for bone metastases were evaluated in one retrospective cohort. Of the 58 patients, the 3-year CRPC-free survival and cancer-specific survival was 75.9% and 91.4% (22).

Toxicities of SBRT included bowel complications, bladder complications, and bone fractures in metastases-directed treatment (23). In this study, no grade 3 or more adverse events were reported. All the adverse events during follow-up were tolerable and controlled through medication. No bone fractures occurred.

Inevitably, this study has several limitations. First, selection bias could not be ruled out. The study cohort included both mHSPC and mCRPC patients, and most of the patients received one or more systemic treatments before or after SBRT. Second, a number of important values such as PSA levels in the follow up, primary tumor stage, and Gleason score of primary tumor were missing. This might lead to underestimation of the tumor aggressiveness and risk of progression. Third, PSMA-imaging, which was considered one of the most sensitive imaging modalities for detection and evaluation of metastatic lesions, has been applied only in recent years in our center. Although approximately half of these patients were diagnosed by PSMA-PET/CT or PSMA-PET/MR, still some patients were evaluated based on enhanced SPECT or MRI. Neglecting micro-metastases may possibly lead to distant progression after SBRT. Last but not least, this study was limited by its retrospective nature.

## Conclusion

SBRT is an effective metastases-directed therapy for oligometastatic PCa with a high local control rate. Toxicity of SBRT could be well-tolerated. Distant metastases and biological progression still occurred after SBRT, implying the importance of systemic treatment in high-risk oligometastatic PCa patients. Still, prospective studies with long-term follow-up results were required to validate the efficacy and safety of SBRT for oligometastatic PCa patients in China.

## Data Availability Statement

The original contributions presented in the study are included in the article/**Supplementary Material**. Further inquiries can be directed to the corresponding authors.

## Ethics Statement

The studies involving human participants were reviewed and approved by the Medical Ethics Committee of Shanghai Changhai Hospital of the Navy Medical University. The patients/participants provided their written informed consent to participate in this study.

## Author Contributions

CX and XZ conceived and designed the experiments and wrote the paper. XJ, YS, MQ, YY, and XW helped to collect and analyze the data. CY helped to analyze the treatment planning. XG and HZ revised the paper. All authors contributed to the article and approved the submitted version.

## Funding

This study is sponsored by the First Affiliated Hospital of Navy Medical University **“**234 Subject Climbing Program**”** (2019YPT004), the First Affiliated Hospital of Navy Medical University **“**Youth Startup Fund**”** (2020QNB10).

## Conflict of Interest

The authors declare that the research was conducted in the absence of any commercial or financial relationships that could be construed as a potential conflict of interest.

## Publisher’s Note

All claims expressed in this article are solely those of the authors and do not necessarily represent those of their affiliated organizations, or those of the publisher, the editors and the reviewers. Any product that may be evaluated in this article, or claim that may be made by its manufacturer, is not guaranteed or endorsed by the publisher.

## Abbreviations

SBRT, Stereotactic body radiation therapy; LC, local control; bPFS, Biochemical progression free survival; PFS, Progression free survival; OS, Overall survival; CR, Complete response; PR, Partial response; SD, Stable disease; PD, Progressive disease; mCRPC, Metastatic castration-resistant prostate cancer; mHSPC, Metastatic hormone-sensitive prostate cancer; ADT, Androgen deprivation therapy; QoL, Quality of life; PCa, Prostate cancer; CT, Computed tomography; GTV, Gross tumor volume; PTV, Planning target volume; AE, Adverse events; PSA, Prostate-specific antigen; PSMA, Prostate-specific membrane antigen; CTCAE, Common Terminology Criteria for Adverse Events; RECIST, Response Evaluation Criteria in Solid Tumors.
