# Supplementary material for: Short-Term Outcomes and Clinical Efficacy of Stereotactic Body Radiation Therapy (SBRT) for Oligometastases of Prostate Cancer in China
Source: Front Oncol. 2022 Apr 28;12:879310. doi: 10.3389/fonc.2022.879310 (PMC9095840; doi:10.3389/fonc.2022.879310)
Supplement: Supplementary file 1 [file Table_1.docx]

**Supplementary material**

**Table S1. Locations and treatment regimen for local progression disease after SBRT**

| **Patient No.** | **Locations** | **Time to progression (month)** | **GTV (ml)** | **Total prescribed dosage (Gy)** | **No. of fractions** |
| --- | --- | --- | --- | --- | --- |
| 1 | T7 | 40.6 | 5.25 | 32 | 5 |
| 2 | Left hip bone | 10.3 | 32.79 | 29 | 5 |
| 3 | T1 | 14.4 | 23.45 | 30 | 5 |
| 4 | L2 | 31.5 | 4.77 | 29 | 5 |
| 4 | L4 | 31.5 | 2.74 | 30 | 5 |
| 5 | T9 | 7.0 | 28.2 | 35 | 7 |

SBRT: Stereotactic body radiation therapy; GTV: Gross tumor volume.
